# Prediction of Aphasia Severity in Patients with Stroke Using Diffusion Tensor Imaging

**DOI:** 10.3390/brainsci11030304

**Published:** 2021-02-27

**Authors:** Jin-Kook Lee, Myoung-Hwan Ko, Sung-Hee Park, Gi-Wook Kim

**Affiliations:** 1 Department of Physical Medicine & Rehabilitation, Jeonbuk National University Medical School, Jeonju 54907, Korea; ljk3269@naver.com (J.-K.L.); mhko@jbnu.ac.kr (M.-H.K.); shpark0130@jbnu.ac.kr (S.-H.P.); 2 Department of Speech-Language Therapy, The Graduate School, Jeonbuk National University, Jeonju 54907, Korea; 3 Research Institute of Clinical Medicine of Jeonbuk National University, Biomedical Research Institute of Jeonbuk National University Hospital, Jeonju 54907, Korea

**Keywords:** aphasia, stroke, white matter, diffusion tensor imaging

## Abstract

This study classified the severity of aphasia through the Western Aphasia Battery and determined the optimal cut-off value for each Language-Related White Matter fiber and their combinations, we further examined the correlations between Language-Related White Matter and Western Aphasia Battery subscores. This retrospective study recruited 64 patients with aphasia. Mild/moderate and severe aphasia were classified according to cut-off Aphasia Quotient score of 51 points. Diffusion tensor imaging and fractional anisotropy reconstructed Language-Related White Matter in multiple fasciculi. We determined the area under the covariate-adjusted receiver operating characteristic curve to evaluate the accuracy of predicting aphasia severity. The optimal fractional-anisotropy cut-off values for the individual fibers of the Language-Related White Matter and their combinations were determined. Their correlations with Western Aphasia Battery subscores were analyzed. The arcuate and superior longitudinal fasciculi showed fair accuracy, the inferior frontal occipital fasciculus poor accuracy, and their combinations fair accuracy. Correlations between Language-Related White Matter parameters and Western Aphasia Battery subscores were found between the arcuate, superior longitudinal, and inferior frontal occipital fasciculi and spontaneous speech, auditory verbal comprehension, repetition, and naming. Diffusion-tensor-imaging-based language-Related White Matter analysis may help predict the severity of language impairment in patients with aphasia following stroke.

## 1. Introduction

Aphasia is defined as the impairment or a loss of language function. Its incidence rate in the acute phase of stroke ranges from 21% to 38%. The clinical characteristics of aphasia correspond to the location and extent of brain injury [1,2,3]. 

The Western Aphasia Battery (WAB) is a standardized test designed to evaluate aphasia occurring as a consequence of stroke, dementia, or other acquired neurological disorders [4,5,6]. Using the WAB, the severity of aphasia can be classified according to the aphasia quotient (AQ): 0–25, very severe aphasia; 26–50, severe aphasia; 51–75, moderate aphasia; and ≥76, mild aphasia [5]. Reportedly, the lower the AQ, the poorer the prognosis of aphasia in patients with stroke [7]. The prognosis of aphasia is also affected by the covariates of the acute/chronic stage and the age of onset of stroke [8,9,10]. WAB is difficult to perform without patient cooperation, which is challenging in cases of severe cognitive impairment and depression [11,12]. 

Diffusion tensor imaging (DTI) is a useful imaging technique that uses the diffusion properties of water to assess the integrity and reconstruction of white matter in three dimensions [13]. In DTI analysis, fractional anisotropy (FA) is a parameter that is used to reveal the degree of directionality of microstructures, such as axons, myelin, and microtubules [14,15,16]. Higher FA values indicate higher neural tract integrity, while lower FA values indicate the disintegration of the neural tract: i.e., more severe white matter damage [14,15,16].

Language-related white matter (LRWM) can be divided into dorsal and ventral pathways. LRWM associated with the dorsal pathway includes the arcuate fasciculus (AF) and superior longitudinal fasciculus (SLF), while the ventral pathway includes the inferior fronto-occipital fasciculus (IFOF), inferior longitudinal fasciculus (ILF), and uncinate fasciculus (UF) [17,18,19]. Recent DTI studies have reported the significant role of the language tracts in aphasia: specifically, dorsal pathway in patient with aphasia, and damage to the AF and SLF was associated with difficulty in repetition [20,21,22,23]. Furthermore, the AF has been found to affect the impairment in rate, informativeness, speech fluency, and naming ability [21,22]. Concerning the relationship between the ventral pathway and aphasia, the IFOF has been found to be related to the linguistic processing of sound to meaning [24,25], the UF has been implicated with word comprehension [26], and the ILF features a significant correlation with the comprehension and repetition of speech [20,27].

Previous research has reported an interaction between the dorsal and ventral pathways in patients with post-stroke aphasia. A disconnection between the AF and IFOF was reported in cases of severe aphasia and decreased AF, IFOF, UF FA, and the disconnection of the dorsal and ventral pathways has been reported. [16,24]. However, few studies considered the correlation of various language fibers in aphasia patients and no study has suggested an optimal cut off value for the severity of aphasia.

This study aimed to classify the severity of aphasia as mild/moderate or severe with the WAB and determine the optimal cutoff value for each DTI-based LRWM fiber or various combination of ventral and dorsal pathways in patients with aphasia following stroke. In addition, we examined the correlation between LRWM and WAB subscores.

## 2. Materials and Methods

### 2.1. Subjects

This retrospective study analyzed the medical records of patients who were diagnosed with stroke and hospitalized in the department of rehabilitation medicine of our hospital between October 2010 and November 2018. The inclusion criteria were as follows: 1st left hemisphere stroke, age between 18–85 years, and available WAB and DTI data. Exclusion criteria included an abnormal oral anatomy and severe medical history at the time of WAB and DTI. There were a total of 64 subjects, 45 men and 19 women, all of whom were right-handed; their mean the duration of education was 10.44 ± 4.28 years (Table 1).

This study was conducted after obtaining approval from the Institutional Review Board (IRB) from Jeonbuk National University Hospital (IRB number: CUH 2019-09-007). The study was conducted in accordance with the Declaration of Helsinki. The need for informed consent was waived due to retrospective nature of the study.

### 2.2. Study Methods

#### 2.2.1. Brain DTI Analysis

In the present study, all images were acquired using a Siemens Verio 3-Tesla MRI scanner (Siemens, Erlangen, Germany) using a single-shot echo-planar imaging sequence with two diffusion sensitizing gradients. To reduce the duration of the scan, we used the Generalized Auto calibrating Partially Parallel Acquisition (GRAPPA); this technique produces superior image quality because it reduces image distortion caused by the echo-planar imaging sequence. Potential image distortion was corrected with an automated image registration program. Imaging parameters were as follows: matrix, 128 mm × 128 mm; field of view, 200 mm^2^; TR, 7000 ms; TE, 105 ms; parallel acquisition factor GRAPPA, 2; EPI factor, 128; b-value, 1000 s/mm^2^; number of excitations, 2. We acquired 46 contiguous slices of 3.0 mm slices parallel to the anterior commissure posterior commissure line with no gap between 30 different diffusion directions.

**Figure 1:**
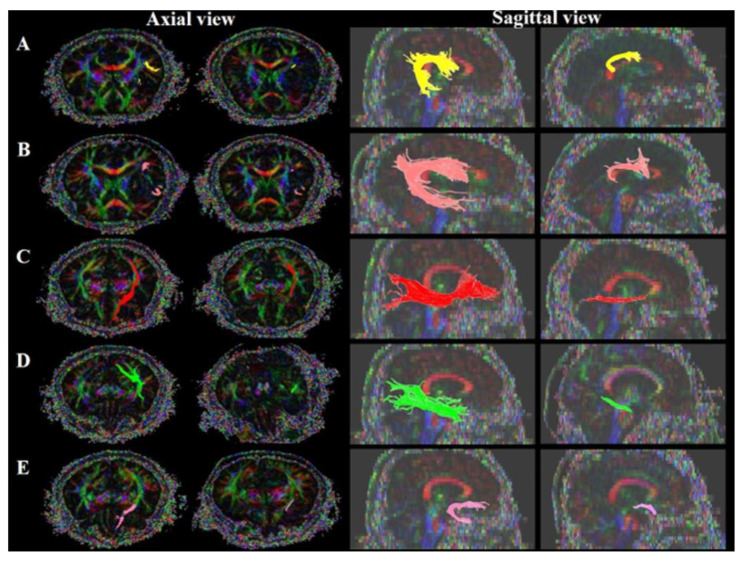
Language-related white matter: (**A**) AF, arcuate fasciculus; (**B**) SLF, superior longitudinal fasciculus (**C**) IFOF, inferior fronto-occipital fasciculus; (**D**) ILF, inferior longitudinal fasciculus; (**E**) UF, uncinate fasciculus.

The AF, SLF, IFOF, UF and ILF of the left hemisphere were reconstructed by using the DTI studio software v.1.02 (CMRM, John Hopkins Medical Institute, Baltimore, MD, USA) to perform fiber tracking with the following parameters: FA threshold of 0.20, a tract turning angle of 60°, and the flip eigenvector as the Y-component. We performed reconstruction by selecting two regions of interest (ROIs) from the brain MRI axial slice: AF, the deep white matter of the posterior parietal portion of superior longitudinal fascicle and the posterior temporal lobe [28]; SLF, the triangular shape just lateral to the corticospinal tract near the anterior horn of the lateral ventricle reticular formation of the medulla and the triangular shape near the posterior horn of the lateral ventricle [29,30,31,32]; IFOF, the external capsule of the coronal section near the anterior horn of the lateral ventricle and around the white matter of the occipital lobe [33,34]; UF, the frontal part of the UF in the coronal plane through the genu of the corpus callosum, just anterior to the anterior horn of the lateral ventricle and the white matter in the coronal plane at the most anterior part of the temporal stem [35,36]; ILF, the posterior border of the white matter of the anterior temporal lobe was defined by the anterior extent of the fibers of the IFOF in order to avoid inclusion of these bundles and around the white matter of the occipital lobe [33,37]). Preserved and disrupted statuses and FA values were determined. We then checked the fiber status in LRWM. Preserved and disrupted statuses could be classified according to the connectivity of the fibers at the site of the stroke lesion [16,28] (Figure 1).

#### 2.2.2. Aphasia Assessment Analysis

The Korean version of the WAB test was used to assess aphasia [30,31]. Subtests of the WAB include spontaneous speech, auditory verbal comprehension, repetition and naming. In the spontaneous speech subtest, the patient responds to questions presented by the examiner and describes a picture stimulus. In the auditory verbal comprehension subtest, the patient answers certain questions with yes or no responses; points to real objects, texts, numbers, colors, objects around the room, and body parts; and responds to stimulus sentences. In the repetition subtest, the patient is instructed to repeat stimulus words of various lengths and complexities that the examiner says. In the naming subtest, the patient is shown objects from various categories and is asked to name them. In addition, the patient is asked to name all animals that the patient can think of in one minute, to complete the latter part of an incomplete stimulus sentence, and to respond to questions. The spontaneous speech, auditory verbal comprehension, repetition, and naming subsets of the WAB account for 20, 10, 10, and 10 points, respectively; hence, the total score of 50 points, which is multiplied by 2 to yield a maximum AQ of 100 points. Although the WAB classifies aphasia severity into four levels according to the AQ score, the present study classified the severity into two levels, which an AQ score of 51 as a cut off between severe or mild/moderate [5,38].

### 2.3. Statistical Analysis

IBM SPSS (statistics for windows, version 24.0. Amonk) software and R (4.0.3) software were used for statistical analysis. Descriptive statistics were performed to assess the demographic data of the subjects. Estimates of the covariate-adjusted ROC curve were used for the area under the covariate-adjusted receiver operating characteristic curve (AAUC), and 95% pointwise posterior credible band of LRWM FA was used to predict aphasia severity. The AAUC was used to evaluate the accuracy of predicting the severity of aphasia with LRWM. AAUCs indicate different levels of accuracy: 0.90–1.00, excellent; 0.80–0.90, good; 0.70–0.80, fair; 0.60–0.70, poor; and 0.50–0.60, fail [39,40]. The receiver operating characteristic (ROC) curves were then used to determine the cut-off values. Finally, partial spearman correlation was performed because the normality test was not sufficient to analyze the correlation when demographic variables were included as covariates. For all statistical tests, the significance threshold was defined as *p* < 0.05. 

## 3. Results

### 3.1. Analysis of Aphasia Severity Using Brain DTI Parameters and Aphasia Assessment Results 

ROC curve analysis yielded the following sensitivity, specificity, and AAUC values regarding the prediction of aphasia severity: the cut-off value of AF FA = 0.380: 0.800, 0.792, and 0.747; the cut-off value of SLF FA = 0.397: 0.725, 0.750, and 0.767, respectively; the cut-off value of IFOF FA = 0.430: 0.700, 0.667, and 0.697, respectively; the cut-off value of UF FA = 0.370: 0.650, 0.625, and 0.579, respectively; and the cut-off of ILF FA = 0.450: 0.500, 0.417, and 0.501, respectively (see Table 2).

The ROC curve analysis yielded the following sensitivity, specificity, and AAUC values for predicting aphasia severity using combinations of two fiber pathways: the cut-off value of AF + SLF FA = 0.7793: 0.750, 0.750, and 0.824, respectively; the cut-off value of AF + IFOF FA = 0.8150: 0.800, 0.708, and 0.688, respectively; the cut-off value of AF + UF FA = 0.7500: 0.750, 0.792, and 0.658, respectively; the cut-off value of AF + ILF FA = 0.8350: 0.675, 0.708, and 0.704, respectively; the cut-off value of SLF + IFOF FA = 0.8250: 0.750, 0.750, and 0.741, respectively; the cut-off value of SLF + UF FA = 0.7405: 0.750, 0.750, and 0.608, respectively; the cut-off value of SLF + ILF FA = 0.8410: 0.700, 0.708, and 0.661, respectively; the cut-off value of IFOF + UF FA = 0.7850: 0.675, 0.625, and 0.633, respectively; the cut-off value of IFOF + ILF FA = 0.8950: 0.625, 0.667, and 0.693, respectively; and the cut-off value of UF + ILF FA = 0.8150: 0.625, 0.625, and 0.567, respectively.

### 3.2. Correlational Analysis of Brain DTI Parameters and Aphasia Assessment Results

Correlations between the subscores of WAB and each LRWM parameter are shown in Table 3.

#### 3.2.1. Spontaneous Speech

Spontaneous speech was positively correlated with AF FA (r = 0.556, *p* < 0.001) and SLF FA (r = 0.463, *p* < 0.001) and IFOF FA (r = 0.334, *p* = 0.010). However, it was not significantly correlated with UF FA (r = 0.252, *p* = 0.056) or with. ILF FA (r = −0.018, *p* = 0.895)

#### 3.2.2. Auditory Verbal Comprehension

Auditory verbal comprehension was positively correlated with AF FA (r = 0.474, *p* < 0.001) and SLF FA (r = 0.468, *p* < 0.001) and IFOF FA (r = 0.397, *p* = 0.002). However, it was not significantly correlated with UF FA (r = 0.241, *p* = 0.068) or ILF FA (r = 0.106, *p* = 0.429).

#### 3.2.3. Repetition

Repetition was positively correlated with AF FA (r = 0.514, *p* < 0.001) and SLF FA (r = 0.477, *p* < 0.001) and IFOF FA (r = 0.263, *p* = 0.047). However, it was not significantly correlated with UF FA (r = 0.183, *p* = 0.168) and ILF FA (r = 0.006, *p* = 0.965).

#### 3.2.4. Naming

Naming was positively correlated with AF FA (r = 0.556, *p* < 0.001) and SLF FA (r = 0.476, *p* < 0.001) and IFOF FA (r = 0.512, *p* < 0.001). However, it was not significantly correlated with UF FA (r = 0.228, *p* = 0.086) and ILF FA (r = 0.156, *p* = 0.273).

## 4. Discussion

LRWM can be divided into dorsal and ventral pathways. The dorsal pathway includes the AF and SLF connecting the temporoparietal region with the frontal premotor region [21,41], while the ventral pathway passes through the extreme capsule to connect the temporal region with the prefrontal region and includes the IFOF, ILF and UF [42,43,44,45].

Recently, the role of the language tract in patients with aphasia has been studied with DTI. Most brain DTI-based studies related to aphasia have reported that the greater the AF damage, the poorer the prognosis and the more serious language impairment [23,46,47,48,49,50,51]. According to previous studies on LRWM, damaged AF and SLF compromised difficulties in repetitive ability and speech fluency [22,41]. Duffau and Gil-Robles reported that checking impaired IFOF and ILF using electrical stimulation was associated with semantic impairment, visual object recognition, and difficulty in reading [24,52]. Harvey reported that impaired UF was related to word comprehension deficits through rs-fMRI, DTI images, and performance on behavioral tasks [26]. 

Patients with severe aphasia present with decreased FA of the AF and IFOF and a disconnection of the dorsal and ventral pathways [41]. In patients with post-stroke aphasia, a correlation was observed between ART, BDAE scales and the FA of the AF, IFOF, and UF. In the acute/chronic stage, patients with severe aphasia present decreased FA of the AF, IFOF, and UF, as well as the disconnection of the dorsal and ventral pathways [53]. 

Few studies have considered the correlation of various language fibers in aphasia patients, and none suggested an optimal cut-off value for the severity of aphasia. Therefore, this study classified the severity of aphasia as mild/moderate or severe according to the AQ of the WAB and determined the optimal cut-off value for each DTI-based LRWM fiber or various combinations of abdominal and dorsal pathways. In addition, we confirmed the correlation between LRWM and WAB subscores.

The AF has is reportedly associated with spontaneous speech, fluency, repetition, naming, sentence comprehension, and overall language impairment [20,21,22,23,54]. AF FA can predict the language functions of aphasic patients and plays an important role in both the receptive language and expressive language areas [55,56]. The present study also determined the cut-off point of LRWM that is optimal for predicting aphasia severity and found the most appropriate cut-off value for AF FA in the single fiber analysis to be 0.380 (sensitivity, 80.0%; specificity, 79.2%; AAUC, 0.747). In addition, two LRWM including the AF also showed high sensitivity, specificity and AAUC, and the FA of the AF was found to be correlated with spontaneous speech, auditory verbal comprehension, repetition and naming. These findings are consistent with those of previous studies [20,21,22,23,54,57].

The SLF is a fiber that constitutes the dorsal pathway of LRWM. The SLF is associated with repetition and expressive and receptive syntactic processing [33,58], as well as with aphasia severity and impaired language performance [20,41]. This study found that the cut-off value of 0.397 for the FA of the SLF in the single fiber analysis had a sensitivity of 72.5%, a specificity of 75.0%, and AAUC of 0.767; these values indicated fair accuracy. The FA of the SLF was correlated with spontaneous speech, auditory verbal comprehension, repetition and naming demonstrating a strong association with aphasia. 

The IFOF is a major direct pathway of the ventral pathway, which is essential for semantic processing. It has been reported that electrical stimulation of the IFOF elicits a semantic error, and because of the correlation between IFOF and AQ, such stimulation also affects language and general cognitive status [24,59,60,61,62]. This study found that the cut-off value of 0.430 for IFOF FA in the single fiber analysis had a sensitivity of 70.0%, a specificity of 66.7% and AAUC of 0.697; while these values indicate poor accuracy, two LRWM including the IFOF showed fair accuracy. The FA of the IFOF was correlated with spontaneous speech, auditory verbal comprehension, repetition and naming demonstrating a strong association with aphasia. 

The UF is a part of the ventral pathway and its function is related to semantic processing [22,56,63]. Studies have shown that damage to the UF leads to semantic processing disorder related to name retrieval [64,65,66,67]. The present study found that the cut-off value of 0.37 for the UF FA in single fiber analysis had a sensitivity of 65.0%, a specificity of 62.5% and an AAUC of 0.579; these values indicated fail accuracy. Correlations of the FA of the UF with WAB subscores were found to be weaker and inconsistent with previous studies. 

The differences between the results of the present study and previous reports may be explained by several factors. First, previous studies used language assessment tools that assessed a specific semantic domain [25,26,68,69], whereas the present study used the WAB: general language assessment tool. Therefore, the WAB may not be sensitive in detecting specific semantic damage [23]. Second, the UF is different from other LRWM in terms of language function lateralization. The AF is lateralized to the left hemisphere, whereas the UF is a bilateral fiber; therefore, loss of language function caused by left hemisphere damage can be more easily compensated in the case of the UF [70,71]. In addition, in the ventral pathway, the IFOF plays an important role in semantic processing, while the UF plays a subsidiary role [72,73]. Therefore, the UF may have shown a weaker correlation with semantic processing.

The ILF is responsible for the processing of semantic information associated with face recognition, reading and naming is involved in visual associative memory, analysis of visual motion, visual-spatial analysis, and attention [27,74,75]. The present study revealed that the cut-off value of 0.450 for ILF FA in the single fiber analysis had a sensitivity of 50.0%, a specificity of 41.7%, and an AAUC of 0.501. These values indicated fail accuracy, and the two LRWM including the ILF showed fail accuracy. Correlations of ILF with WAB subscores were weaker and inconsistent with previous studies [27,63,74]. However, the ILF is more associated with visual object recognition [52,76], language, comprehension, and naming [20,27,73,76]. Therefore, at an insignificant level, the ILF showed a stronger correlation with AVC and naming subscores that utilize visual stimulus [5] than with other subscores of the WAB. The difference between the results of the present study and previous reports may be attributable to the different functions of each segment of the LRWM [43,77]. In particular, the middle and posterior segments of the ILF, which is situated beneath the middle and inferior portions of the left temporal lobe, is strongly associated with word and sentence comprehension [49,73,78]; the anterior portion of the ILF, which is situated relatively closer to the insular lobe, is associated with naming [60]. Therefore, it may be difficult to determine the functional relevance of the whole fiber [74].

According to ROC analysis, the AF and SLF in the dorsal pathway achieved fair accuracy as single fibers in the in the AAUC analysis, and the IFOF in the ventral pathway yielded an AAUC of 0.697. The combinations of the AF and ILF and the SLF and IFOF achieved fair accuracy. Associations between LRWM parameters and WAB subscores showed correlations between the AF and SLF in the dorsal pathway and IFOF in the ventral pathway in SS, AVC, repetition, and naming. 

Our study is subject to some limitations. The first limitation of the current study is its not having analyzed lesions according to size differences and location. Second, there was no control group for comparison. Third, the analysis included a small number of patients, and the results were not obtained using specific language domain assessment tools or by analyzing extensive data. In future research, we aim to expand on this work and consider including a control group for comparison, assessing the location and size of lesions, increasing number of patients, incorporating into our analysis the date of injury, and assessment tool.

## 5. Conclusions

In conclusion, we determined the optimal cut-off values and AACU of LRWM fibers according to the severity of aphasia and confirmed the correlation between LRWM and WAB subscores. The analysis of LRWM using DTI may be helpful for predicting the severity of language impairment in patients with aphasia following stroke.

## Figures and Tables

**Table 1:** Characteristics of the patients

Variables	Aphasic Patients
Age (years)	56.52 ± 15.48
Sex (M/F)	45 (70.3%)/19 (29.7%)
Infarction (n/%)/hemorrhage (n/%)	10 (15.6%)/54 (84.4%)
Education (years)	10.44 ± 4.28
Time from onset to WAB (days)	25.09 ± 15.23
Time from onset to DTI evaluation (days)	24.61 ± 13.81

Abbreviations: DTI, diffusion tensor imaging; WAB, Western Aphasia Battery.

**Table 2:** ROC curve analysis according to aphasia severity.

	Tracts	Cut-Off Value	Sensitivity	Specificity	AAUC (φ)
Single fiber	AF FA	0.380	0.800	0.792	0.747 (0.529, 0.914)
	SLF FA	0.397	0.725	0.750	0.767 (0.621, 0.881)
	IFOF FA	0.430	0.700	0.667	0.697 (0.539, 0.833)
	UF FA	0.370	0.650	0.625	0.579 (0.405, 0.738)
	ILF FA	0.450	0.500	0.417	0.501 (0.315, 0.686)
	AF + SLF FA	0.7793	0.750	0.750	0.824 (0.688, 0.918)
	AF + IFOF FA	0.8150	0.800	0.708	0.688 (0.470, 0.876)
	AF + UF FA	0.7500	0.750	0.792	0.658 (0.507, 0.795)
Two fibers	AF + ILF FA	0.8350	0.675	0.708	0.704 (0.513, 0.865)
	SLF + IFOF FA	0.8250	0.750	0.750	0.741 (0.587, 0.870)
	SLF + UF FA	0.7405	0.750	0.750	0.608 (0.400, 0.800)
	SLF + ILF FA	0.8410	0.700	0.708	0.661 (0.468, 0.833)
	IFOF + UF FA	0.7850	0.675	0.625	0.633 (0.414, 0.826)
	IFOF + ILF FA	0.8950	0.625	0.667	0.693 (0.522, 0.837)
	UF + ILF FA	0.8150	0.625	0.625	0.567(0.396, 0.730)

Abbreviations: AAUC, area under the covariate-adjusted ROC curve; AF, arcuate fasciculus; SLF, superior longitudinal fasciculus; IFOF, inferior fronto-occipital fasciculus; ILF, inferior longitudinal fasciculus; UF, uncinate fasciculus; FA, fractional anisotropy; φ, 95% pointwise posterior credible band.

**Table 3:** Correlations between LRWM parameters and WAB subscores.

	SS	AVC	Repetition	Naming
**AF FA**	r = 0.556	r = 0.474	r = 0.514	r = 0.556
	*p* < 0.001 **	*p* < 0.001 **	*p* < 0.001 **	*p* < 0.001 **
**SLF FA**	r = 0.463	r = 0.468	r = 0.477	r = 0.476
	*p* < 0.001 **	*p* < 0.001 **	*p* < 0.001 **	*p* < 0.001 **
**IFOF FA**	r = 0.334	r = 0.397	r = 0.263	r = 0.512
	*p* = 0.010 *	*p* = 0.002 *	*P*= 0.047 *	*p* < 0.001 **
**UF FA**	r = 0.252	r = 0.241	r = 0.183	r = 0.228
	*p* = 0.056	*p* = 0.068	*p* = 0.168	*p* = 0.086
**ILF FA**	r = -0.018	r = 0.106	r = 0.006	r = 0.156
	*p* = 0.895	*p* = 0.429	*p* = 0.965	*p* = 0.273

Abbreviations: SS, spontaneous speech; AVC, auditory verbal comprehension; AF, arcuate fasciculus; SLF, superior longitudinal fasciculus; IFOF, inferior fronto-occipital fasciculus; ILF, inferior longitudinal fasciculus; UF, uncinate fasciculus; FA, fractional anisotropy. * *p* < 0.05, ** *p* < 0.001.

## Data Availability

The data used to support the findings of this study are available from the corresponding author upon request.
